# High-Index-Faceted Ni_3_S_2_ Branch Arrays as Bifunctional Electrocatalysts for Efficient Water Splitting

**DOI:** 10.1007/s40820-019-0242-8

**Published:** 2019-02-05

**Authors:** Shengjue Deng, Kaili Zhang, Dong Xie, Yan Zhang, Yongqi Zhang, Yadong Wang, Jianbo Wu, Xiuli Wang, Hong Jin Fan, Xinhui Xia, Jiangping Tu

**Affiliations:** 1 State Key Laboratory of Silicon Materials, Key Laboratory of Advanced Materials and Applications for Batteries of Zhejiang Province, and Department of Materials Science and Engineering, Zhejiang University, Hangzhou, 310027 People’s Republic of China; 2 Guangdong Engineering and Technology Research Center for Advanced Nanomaterials, School of Environment and Civil Engineering, Dongguan University of Technology, Dongguan, 523808 People’s Republic of China; 3 School of Physical and Mathematical Sciences, Nanyang Technological University, Singapore, 637371 Singapore; 4 School of Engineering, Nanyang Polytechnic, Singapore, 569830 Singapore; 5 Zhejiang Provincial Key Laboratory for Cutting Tools, Taizhou University, Taizhou, 318000 People’s Republic of China

**Keywords:** Nickel sulfide, Core/branch arrays, Porous film, Bifunctional electrocatalysts, Electrochemical water splitting, Oxygen evolution reaction (OER), Hydrogen evolution reaction (HER)

## Abstract

For efficient electrolysis of water for hydrogen generation or other value-added chemicals, it is highly relevant to develop low-temperature synthesis of low-cost and high-efficiency metal sulfide electrocatalysts on a large scale. Herein, we construct a new core–branch array and binder-free electrode by growing Ni_3_S_2_ nanoflake branches on an atomic-layer-deposited (ALD) TiO_2_ skeleton. Through induced growth on the ALD-TiO_2_ backbone, cross-linked Ni_3_S_2_ nanoflake branches with exposed {$\bar{2}10$**}** high-index facets are uniformly anchored to the preformed TiO_2_ core forming an integrated electrocatalyst. Such a core–branch array structure possesses large active surface area, uniform porous structure, and rich active sites of the exposed {$\bar{2}10$} high-index facet in the Ni_3_S_2_ nanoflake. Accordingly, the TiO_2_@Ni_3_S_2_ core/branch arrays exhibit remarkable electrocatalytic activities in an alkaline medium, with lower overpotentials for both oxygen evolution reaction (220 mV at 10 mA cm^−2^) and hydrogen evolution reaction (112 mV at 10 mA cm^−2^), which are better than those of other Ni_3_S_2_ counterparts. Stable overall water splitting based on this bifunctional electrolyzer is also demonstrated.

**Electronic supplementary material:**

The online version of this article (10.1007/s40820-019-0242-8) contains supplementary material, which is available to authorized users.

## Highlights

- TiO_2_@Ni_3_S_2_ core/branch arrays are constructed via a low-temperature sulfurization.
- Highly active {$\bar{2}10$} high-index facet of Ni_3_S_2_ is exposed for both oxygen evolution reaction (OER) and hydrogen evolution reaction (HER).
- Remarkable bifunctional electrocatalytic activity is observed for both HER and OER.

**Electronic supplementary material:**

The online version of this article (10.1007/s40820-019-0242-8) contains supplementary material, which is available to authorized users.

## Graphical abstract

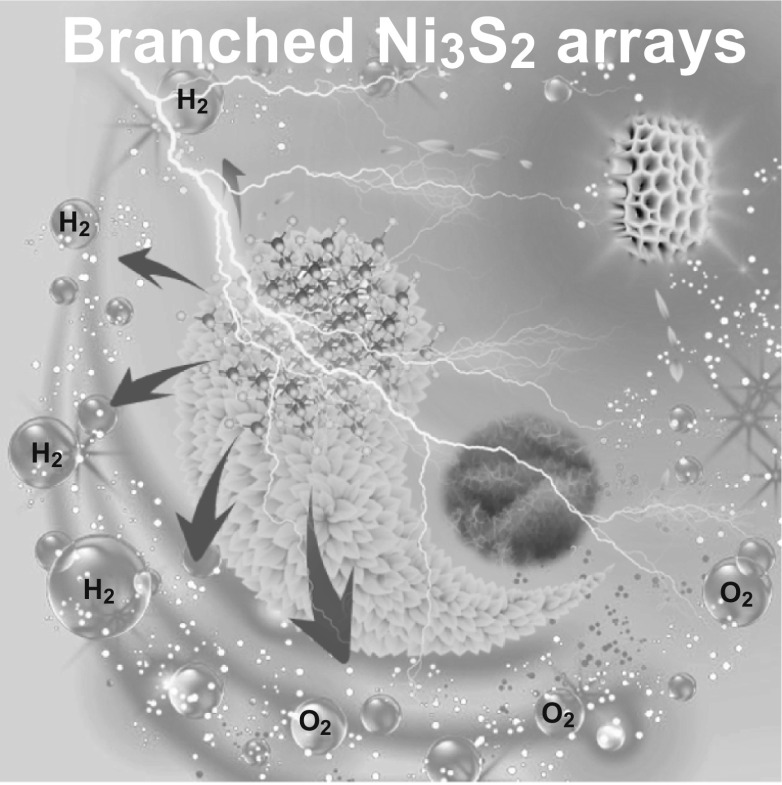

## Introduction

Production of hydrogen/oxygen fuels through electrochemical water splitting is considered one of the most efficient green technologies, although large-scale synthesis of cost-effective electrocatalysts used in this process still remains a huge challenge [1–5]. Platinum (Pt)/Pt-based alloys and iridium/ruthenium oxides (IrO_2_/RuO_2_) are regarded as the most efficient electrocatalysts for electrochemical hydrogen evolution reaction (HER) and oxygen evolution reaction (OER), respectively [6–10]. However, their high cost and compromised stability as well as the low earth abundance of these metals impede their widespread application [11–15]. Therefore, it is highly desirable to fabricate alternative noble-metal-free and durable electrocatalysts for both OER and HER systems. Although transition metal oxides and hydroxides (NiO, CoO, Ni(OH)_2_, etc.) [16] are being widely investigated, they mostly have intrinsically low electrical conductivity and their composites with carbon additives should be prepared to improve the electrical conductivity. Metal sulfides, such as nickel sulfide (Ni_3_S_2_), are more attractive candidates for electrochemical water splitting, owing to their intrinsic high conductivity, rich catalytic activity, and superior electrochemical stability when applied in HER/OER [17, 18]. Currently, a wide range of nanostructured Ni_3_S_2_ (such as Fe-doped Ni_3_S_2_ [19] and nanorods [20]) and composites (e.g., Ni_3_S_2_ nanosheets/Ni [21], Ni_3_S_2_ nanotube/Ni [18]) has been prepared by different methods. They demonstrate improved performance in HER or OER owing to increased exposure of the active sites and boosted ion/electron transfer. Despite these efforts, the overall water-splitting activity of the same Ni_3_S_2_-based catalysts for both HER and OER has been rarely reported. In addition, the aforementioned Ni_3_S_2_ electrocatalysts are usually synthesized via chemical vapor deposition (CVD) and hydrothermal methods. However, these methods require high-temperature treatment or the use of polluted thiourea or trithiocyanuric acid. Moreover, the high-temperature treatment may cause the coverage or loss of the active sites of Ni_3_S_2_ [22–25]. In this context, a facile and green low-temperature synthesis method for Ni_3_S_2_ electrocatalysts is highly desirable.

Low-temperature (< 100 °C) sulfurization using a Na_2_S solution is a green way for the large-scale synthesis of nanostructured metal sulfides owing to easy processing, high efficiency, and cost-effectiveness. Moreover, this method is particularly suitable for the direct synthesis of metal sulfides arrays with tailored nanostructures. Meanwhile, it has been demonstrated that a high-index-faceted Ni_3_S_2_ nanosheet could have superior HER activity owing to possible synergistic catalytic effects arising from the nanosheet array and the exposed {$\bar{2}10$} high-index facets [26]. Inspired by these encouraging results, we set out to employ a low-temperature synthesis route to produce Ni_3_S_2_ nanoarrays with preferentially exposed {$\bar{2}10$} high-index facets as a binder-free electrocatalyst. In addition, in order to further increase the areal load of the active material, we aimed to grow the Ni_3_S_2_ arrays as branches on a conductive scaffold to form a core–branch array structure, instead of directly depositing them on carbon cloth.

Herein, we report a facile low-temperature (< 100 °C) sulfurization strategy to synthesize large-scale TiO_2_@Ni_3_S_2_ core/branch arrays as a binder-free electrode for a water-splitting electrolyzer in an alkaline solution. An induced growth process for growing Ni_3_S_2_ nanobranch on a TiO_2_ core obtained by atomic layer deposition (ALD) is proposed, which leads to the in situ growth of {$\bar{2}10$} high-index facets of Ni_3_S_2_. The as-prepared TiO_2_@Ni_3_S_2_ core/branch arrays possess large active areas, uniform porous structures, and rich active sites of the exposed {$\bar{2}10$} high-index facet. These features lead to substantial enhancements in HER and OER activities compared to those of most of the reported Ni_3_S_2_-based catalysts. Low overpotentials and fast kinetics as well as superior long-term durability of TiO_2_@Ni_3_S_2_ core/branch arrays are demonstrated. A low-water-splitting voltage of 1.58 V at 10 mA cm^−2^ is obtained upon using the TiO_2_@Ni_3_S_2_ array electrode as both a cathode and an anode. Our new electrode design strategy paves a green way for the construction of large-scale nickel sulfides with high electrocatalytic efficiency for electrochemical energy storage and conversion applications.

## Experimental

### Material Synthesis

In the first step, Ni_2_(OH)_2_CO_3_ nanosheet arrays were obtained by a one-step hydrothermal method using commercial nickel foam as the substrate. For this, Ni(NO_3_)_2_ (0.9 g), NH_4_F (0.23 g), and urea (0.9 g) were dissolved in 75 mL of deionized (DI) water to form a reaction solution. Then, the solution was transferred to a Teflon-lined steel autoclave, and the autoclave was placed in an oven at 120 °C for 8 h. After natural cooling, the sample was rinsed thoroughly with DI water.

In order to synthesize TiO_2_@Ni_2_(OH)_2_CO_3_ nanoflake arrays, the prepared Ni_2_(OH)_2_CO_3_ nanosheet arrays were placed in an ALD reactor (ALD PICOSUN P-300F) along with TiCl_4_ and H_2_O as the Ti and O source, respectively. Then, TiO_2_ was deposited at 120 °C for 140 cycles. Argon was used as the carrier gas. The final step was the sulfurization process. Typically, the obtained TiO_2_@Ni_2_(OH)_2_CO_3_ nanoflake array samples were transferred to a 0.1 M Na_2_S solution and heated at 90 °C for 9 h. After natural cooling and rinsing with DI water, the TiO_2_@Ni_3_S_2_ core/branch arrays were obtained. For comparison, Ni_3_S_2_ nanoflake arrays were also synthesized by the direct sulfurization of the Ni_2_(OH)_2_CO_3_ nanosheet arrays on nickel foam (without the ALD TiO_2_ step) using the same sulfurization conditions mentioned above.

### Material Characterization

Morphologies and microstructures of all samples were investigated using a field-emission scanning electron microscope (FESEM, Hitachi SU8010) and a transmission electron microscope (TEM, JEOL 2100F). The crystal structure was characterized by X-ray diffraction (XRD) with Cu Kα radiation (RigakuD/Max-2550). Raman spectra were collected using a Renishaw-inVia Raman microscope with 514-nm laser excitation. X-Ray photoelectron spectroscopy was performed on an ESCALAB_250Xi X-Ray photoelectron spectrometer with an Al Kα source. Specific surface area distributions were obtained using a porosity instrument (BET, JW-BK112).

### Electrochemical Measurements

HER and OER experiments were conducted using an electrochemical workstation (CH Instrument 660D) with a standard three-electrode setup at room temperature; the as-prepared samples, carbon rod (*D* = 8 mm), and saturated calomel electrode were used as the working electrode, counter electrode, and reference electrode, respectively. A 1 M KOH solution was used as the electrolyte for both HER and OER tests. All potentials in this work are referred to the reversible hydrogen electrode. All measurements were first carried out following a cyclic voltammetry (CV) test at 100 mV s^−1^ to stabilize the current. Linear sweep voltammetry (LSV) tests were performed at a scan rate of 5 mV s^−1^. The Tafel plots of the samples were obtained from the LSV curves acquired with a scan rate of 1 mV s^−1^. Electrochemical impedance spectroscopy (EIS) was performed at the polarization voltage being indexed to the current density of 10 mA cm^−2^, in the frequency range of 100 kHz to 50 mHz with an AC amplitude of 10 mV. The stability test was carried out after 10,000 CV cycles. These results were obtained by iR compensation. The overall water splitting was performed in a two-electrode catalyzer, where two pieces of TiO_2_@Ni_3_S_3_ samples with a geometric area of 1 cm^2^ were used as the electrodes for HER and OER.

## Results and Discussion

### Physicochemical Properties of TiO_2_@Ni_3_S_2_ Core/Branch Arrays

**Fig. 1:**
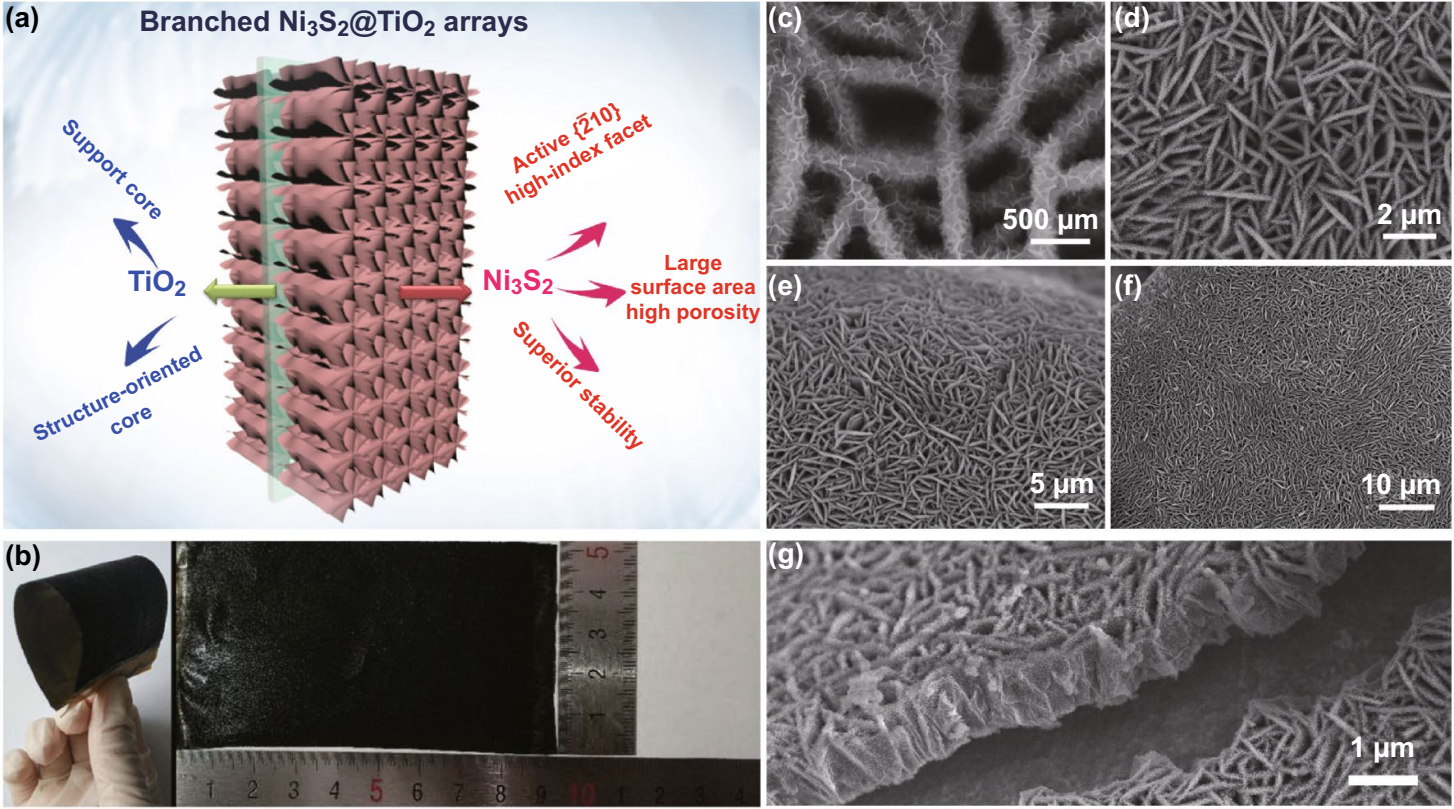
**a** Schematic illustration of the core/branch structure of the TiO_2_@Ni_3_S_2_ arrays. **b** Optical photograph of the sample. **c**–**g** SEM images of the TiO_2_@Ni_3_S_2_ core/branch arrays

The core/branch structure of the TiO_2_@Ni_3_S_2_ arrays is schematically illustrated in Fig. 1a. Ni_2_(OH)_2_CO_3_ nanoflake arrays were synthesized on commercial nickel foam via a standard hydrothermal process (see details in Sect. 2.1). A TiO_2_ layer with 10 nm thickness was deposited on the surface of the Ni_2_(OH)_2_CO_3_ nanoflakes using a simple ALD method. The obtained TiO_2_@Ni_2_(OH)_2_CO_3_ arrays were converted to TiO_2_@Ni_3_S_2_ core/branch arrays by immersing them into a Na_2_S solution and heated. We applied this unique-structured material as electrocatalyst and propose the following advantages in enhancing the HER and OER:

1. Branched Ni_3_S_2_ nanoflakes possess a high surface area and higher porosity than those of the pure Ni_3_S_2_ nanoflakes grown directly on Ni foam. Further, the open structure of the interconnected nanoflakes will facilitate ion diffusion and H_2_/O_2_ detachment during the HER/OER processes. This is particularly beneficial for large-current electrocatalysis.
2. The ALD-TiO_2_ skeleton not only serves a mechanical support for the Ni_3_S_2_ branch, but also induces the nucleation for the directional growth of Ni_3_S_2_. Without the ALD-TiO_2_ skeleton, no Ni_3_S_2_-branch can be formed. The TiO_2_ and Ni_3_S_2_ act synergistically to provide better mechanical stability and enhanced specific surface area and larger porosity [27, 28].
3. One important feature of this unique branched Ni_3_S_2_ nanoflakes is the exposure of their highly active {$\bar{2}10$} high-index facets, which can further improve the HER/OER activities leading to a lower overpotential and Tafel slope.

The morphological evolution of the samples at different stages of the synthesis is revealed by the SEM images (see Fig. S1). The hydrothermally synthesized Ni_2_(OH)_2_CO_3_ nanoflakes with thicknesses between 40 and 60 nm are found aligned vertically on the nickel foam surface, forming an architecture with a porous network (Fig. S1a, b). After the ALD of TiO_2_, the twisted nanoflakes of Ni_2_(OH)_2_CO_3_ smoothened to form TiO_2_@Ni_2_(OH)_2_CO_3_ core/shell arrays. Further, the thickness of the TiO_2_@Ni_2_(OH)_2_CO_3_ core/shell arrays increased to 50–70 nm. However, the 3D porous structure is still preserved, which is not surprising since the ALD generally results in a uniform and conformal deposition of a smooth thin film of amorphous TiO_2_ (Fig. S1c, d). However, after the final sulfurization in Na_2_S solution at 90 °C, the morphology changed radically; the previous core/shell structure of TiO_2_@Ni_2_(OH)_2_CO_3_ transformed into a new type of branched structure of TiO_2_@Ni_3_S_2_. It is observed that the TiO_2_@Ni_3_S_2_ sample is black and the display area is ~ 45 cm^2^. This process can be easily adapted for large-scale production (Fig. 1b). Meanwhile, the internal TiO_2_ core is homogeneously coated by the cross-linked Ni_3_S_2_ nanoflake shell with 10–15 nm thickness (Fig. 1c, f). Furthermore, the porous morphology remained well preserved in the TiO_2_@Ni_3_S_2_ core/branch arrays. These unique porous structural features provide a number of tunnels to boost electron/ion transfer. As shown in Fig. 1g, the TiO_2_@Ni_3_S_2_ core/branch arrays grew quasi-vertically with respect to the substrate with a height of ~ 1 μm.

**Fig. 2:**
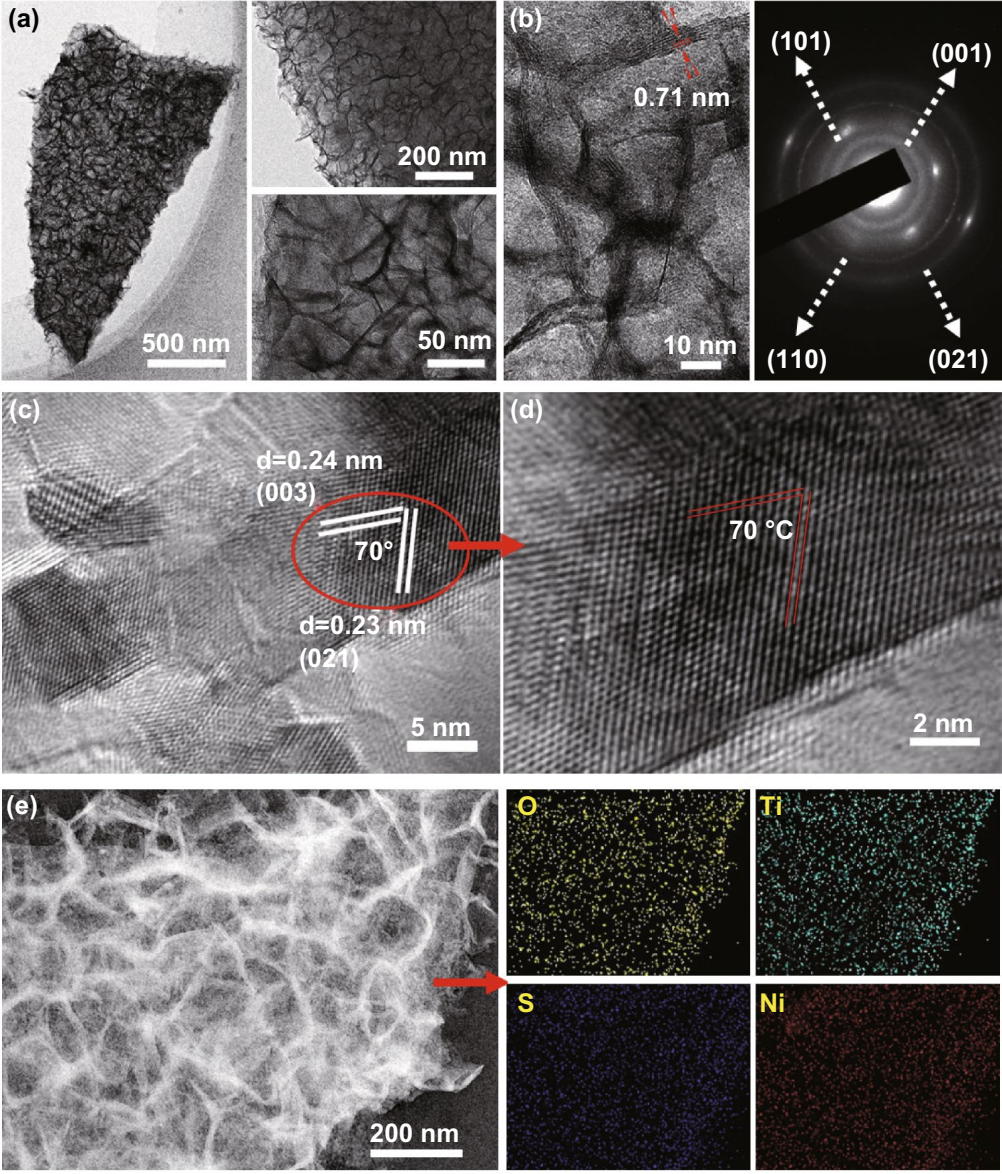
**a**–**d** TEM-HRTEM images and SAED pattern of TiO_2_@Ni_3_S_2_ core/branch arrays. **e** EDS elemental maps of O, S, Ni, and Ti in TiO_2_@Ni_3_S_2_ core/branch arrays

The branched microstructure of the TiO_2_@Ni_3_S_2_ arrays was also explored by TEM observation. The Ni_2_(OH)_2_CO_3_ nanoflake presents a thin and smooth appearance (Fig. S2a). The measured interplanar *d*-spacing of Ni_2_(OH)_2_CO_3_ is about 0.26 nm, which corresponds well with that of the (−201) plane of Ni_2_(OH)_2_CO_3_ (JCPDS 35-0501) (Fig. S2b) [29]. After the ALD of TiO_2_, the Ni_2_(OH)_2_CO_3_ is completely coated with a thin layer of TiO_2_ with ~ 10 nm thickness (Fig. S2c, d), forming a TiO_2_@Ni_2_(OH)_2_CO_3_ core/shell structure. Additionally, the thin TiO_2_ is amorphous in nature and the interplanar *d*-spacing of 0.26 nm is still noticed for Ni_2_(OH)_2_CO_3_. As for the TiO_2_@Ni_3_S_2_ sample, the pristine TiO_2_@Ni_2_(OH)_2_CO_3_ core/shell structure changed to core/branch array, in which the TiO_2_ core is homogeneously covered by cross-linked Ni_3_S_2_ nanoflakes (Fig. 2a). A clear interplanar *d*-spacing of ~ 0.71 nm is observed, which may be due to the expansion of the *c* axis of Ni_3_S_2_. In addition, the selected area electron diffraction (SAED) pattern shows polycrystalline diffraction rings of the TiO_2_@Ni_3_S_2_ sample (Fig. 2b), which is in good agreement with the (001), (101), (110), and (021) planes of Ni_3_S_2_.

HRTEM investigation was performed along the [100] zone axis of Ni_3_S_2_, and interestingly, the interplanar *d*-spacing of 0.24 and 0.23 nm matched well with the (003) and (021) planes of the hexagonal Ni_3_S_2_ phase (JCPDS 44-1418). Further, the angle between the (003) and (021) facets is approximately 70°, which corresponds to the theoretical value of 70.8° (Fig. 2c, d). This implies that the exposed facets of the Ni_3_S_2_ nanoflakes are {$\bar{2}10$} high-index facets. According to a previous report by Fang et al. [26], this facet shows superior catalytic performance. Energy-dispersive X-ray spectroscopy (EDS) maps (Fig. 2e) confirm the presence and uniform distribution of O, S, Ni, and Ti in the TiO_2_@Ni_3_S_2_ arrays.

In our experiment, the ALD-TiO_2_ skeleton serves as a nucleation core for the directional growth of Ni_3_S_2_ nanosheets. Without the ALD-TiO_2_ skin, no Ni_3_S_2_ branch can be formed. Comparatively, only common Ni_3_S_2_ nanoflake arrays are formed in the absence of the TiO_2_ layer support (Fig. S3a, b). Interestingly, exposed {$\bar{2}10$} facets are also found in common Ni_3_S_2_ nanosheet arrays, indicating that the low-temperature sulfurization method is favorable for the growth of the high-index {$\bar{2}10$} facet, which is also confirmed by TEM and XRD (Fig. S3c, d). During the sulfurization process, the Ni ions would diffuse outward and combine with sulfur-containing groups (S^2−^, HS^−^, etc.) along the outer surface of ALD-TiO_2_ to form Ni_3_S_2_ nanocrystal nuclei. This outward diffusion process might be related to the Ostwald ripening effect, in which the energy of the interior is higher than that on outer surface. Ni_3_S_2_ species are spontaneously attached to the ALD-TiO_2_ surface, which induces the growth of active nucleation centers and decreases the interfacial energy barrier for the self-assembly of the Ni_3_S_2_ nanoflake branches.

To further demonstrate the benefits of the core/branch arrays, the specific surface area was determined by BET (Fig. S4). The common Ni_3_S_2_ arrays and TiO_2_@Ni_3_S_2_ branch nanosheet arrays show specific surface areas of 1.594 and 4.623 m^2^ g^−1^, respectively, implying that branching leads to significantly increased surface area. Furthermore, the branched nanoflakes are beneficial in that they provide increased exposed active area/sites, leading to increased utilization of the active Ni_3_S_2_ catalyst.

**Fig. 3:**
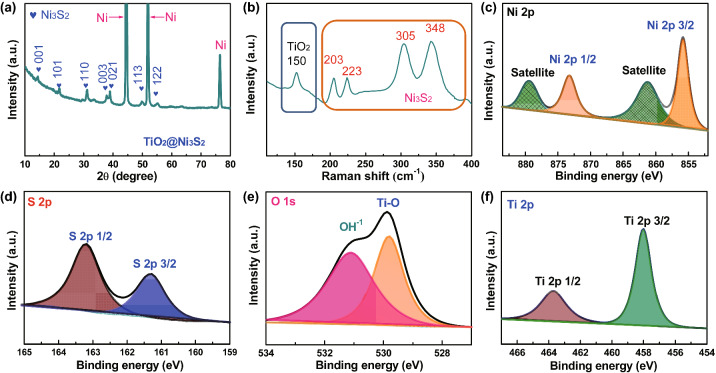
**a** XRD patterns, **b** Raman spectrum, **c** core-level Ni 2*p* XPS spectra, **d** core-level S 2*p* XPS spectra, **e** O 1*s* XPS spectra, and **f** Ti 2*p* XPS spectra of TiO_2_@Ni_3_S_2_ core/branch arrays

In order to identify the phase and composition of the final product, XRD, Raman spectroscopy, and XPS were carried out. Figures S5 and 3a show the typical XRD patterns of Ni_2_(OH)_2_CO_3_, TiO_2_@Ni_2_(OH)_2_CO_3_, and TiO_2_@Ni_3_S_2_. Except for the diffraction peaks of Ni foam, other diffraction peaks in the XRD pattern of Ni_2_(OH)_2_CO_3_ correspond well with those of the monoclinic Ni_2_(OH)_2_CO_3_ phase (JCPDS 35-0501), suggesting the high crystallinity of Ni_2_(OH)_2_CO_3_. For the TiO_2_/Ni_2_(OH)_2_CO_3_ arrays, the diffraction peaks of Ni_2_(OH)_2_CO_3_ can be still detected, but the strength of them decreases. No peaks of the TiO_2_ are noticed, indicating the amorphous nature of the TiO_2_ skeleton (Fig. S5). After the low-temperature sulfurization using the Na_2_S solution, the diffraction peaks of Ni_2_(OH)_2_CO_3_ disappear, and other diffraction peaks that can be indexed well with the Ni_3_S_2_ phase (JCPDS 44-1418) are observed apart from the peaks of metallic Ni foam substrate, demonstrating that the as-obtained TiO_2_@Ni_3_S_2_ sample is of high purity [30]. It is worth noting that the strong diffraction peaks of (021) and (003) plane can be observed clearly (Fig. 3a). Meanwhile, the Raman analysis further confirms the formation of the TiO_2_@Ni_3_S_2_ phase. The TiO_2_@Ni_3_S_2_ arrays show five characteristic peaks at ~ 203, 223, 305, and 348 cm^−1^, which match well with those of the Ni_3_S_2_ phase. The characteristic peak at ~ 150 cm^−1^ could be indexed well with that of amorphous TiO_2_ (Fig. 3b) [1], further manifesting the successful preparation of TiO_2_@Ni_3_S_2_ core/branch arrays. This conclusion is also supported by XPS results. Figure 3c shows the high-resolution Ni 2*p* spectra of the TiO_2_@Ni_3_S_2_ sample. Two main core levels (Ni 2*p*3/2 and Ni 2*p*1/2) that are characteristic of the Ni state in Ni_3_S_2_ are located at 873.28 and 855.78 eV, respectively [31]. As for the S 2*p* spectra, two characteristic peaks are detected at 163.28 eV (S 2*p*1/2) and 161.28 eV (S 2*p*3/2) corresponding to S^2−^ (Fig. 3d) [32]. Moreover, the presence of TiO_2_ in the TiO_2_@Ni_3_S_2_ core/branch arrays is also confirmed by Ti 2*p* and O 1*s* spectra (Fig. 3e, f). Two core-level peaks are located at 529.8 and 531.1 eV in the O 1*s* spectra, which could be indexed well with Ti–O and O–H bonds, respectively [33, 34]. Ti 2*p*1/2 (463.8 5 eV) and Ti 2*p*3/2 (458.0 eV), the characteristic peaks of TiO_2_ are observed in the Ti 2*p* spectra. The presence of O–H bond may be due to surface oxidation of Ni_3_S_2_ [35–37]. All these results mutually confirm the successful fabrication of TiO_2_@Ni_3_S_2_ core/branch arrays via our powerful low-temperature sulfurization strategy.

### Electrocatalytic Properties of TiO_2_@Ni_3_S_2_ Core/Branch Arrays

**Fig. 4:**
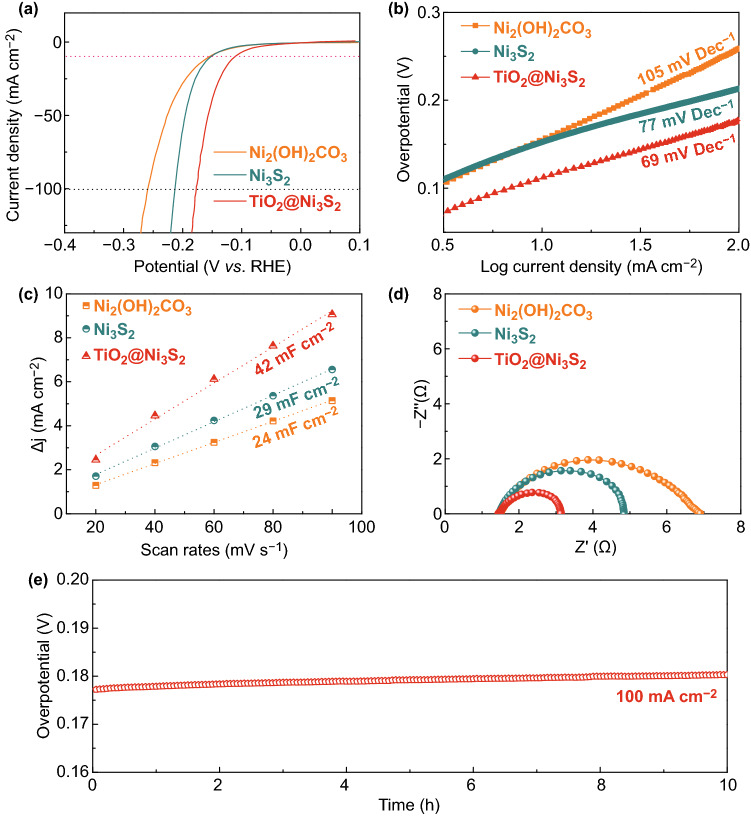
Evaluation of the HER performance and comparison of Ni_2_(OH)_2_CO_3_, Ni_3_S_2_, and TiO_2_@Ni_3_S_2_ electrodes: **a** LSV curves, **b** Tafel plots, **c** current density as a function of scan rate, and **d** Nyquist plots of the Ni_2_(OH)_2_CO_3_, Ni_3_S_2_, and TiO_2_@Ni_3_S_2_ electrodes. **e** Electrochemical stability of the TiO_2_@Ni_3_S_2_ electrode at a current density of 100 mA cm^−2^

The electrocatalytic activity of the three samples (Ni_2_(OH)_2_CO_3_, Ni_3_S_2_, and TiO_2_@Ni_3_S_2_ electrodes) was studied using a three-electrode system in a 1 M KOH solution [38–41]. As presented in Fig. 4a, significantly, the TiO_2_@Ni_3_S_2_ electrode displays the best HER activity with the smallest overpotential (112 mV at the current density of 10 mA cm^−2^), better than that of the Ni_2_(OH)_2_CO_3_ nanoflake arrays (154 mV) and Ni_3_S_2_ (149 mV) nanoflake arrays at the current density of 10 mA cm^−2^. Meanwhile, the TiO_2_@Ni_3_S_2_ core/branch arrays also show a large current density with the lowest overpotential (177 mV at the current density of 100 mA cm^−2^), superior to those of the Ni_2_(OH)_2_CO_3_ (259 mV) and Ni_3_S_2_ (213 mV) counterparts. Additionally, the enhanced HER performance is further confirmed by the Tafel slopes (Fig. 4b) derived from the previous LSV curves. Obviously, the Ni_2_(OH)_2_CO_3_ and Ni_3_S_2_ electrodes show large Tafel slopes (105 and 77 mV/decade), while the TiO_2_@Ni_3_S_2_ electrode exhibits a substantially lower Tafel slope of 69 mV per decade. It is well accepted that a lower Tafel slope implies a faster HER rate. As a result, the TiO_2_@Ni_3_S_2_ electrode leads to the fastest HER process.

Furthermore, the HER performance of our designed high-index faceted Ni_3_S_2_ nanoflake arrays is also excellent. It is well known that HER involves three principal steps including Tafel (30 mV per decade) reactions, Heyrovsky (40 mV per decade), and Volmer (120 mV per decade) mechanisms [42]. Hence, it can be inferred that the HER with Ni_3_S_2_ and TiO_2_@Ni_3_S_2_ electrodes in the alkaline water splitting is based on the Volmer mechanism. Simultaneously, the long-cycle durability of electrocatalysts plays an important role in practical application. The electrochemical stability test of the TiO_2_@Ni_3_S_2_ arrays was carried out continuously at the scan rate of 50 mV s^−1^ for 10,000 CV cycles. The LSV curves of the TiO_2_@Ni_3_S_2_ electrode before and after 10,000 CV cycles of electrolysis nearly overlap with each other, suggesting the excellent stability of TiO_2_@Ni_3_S_2_ electrode (Fig. S6a).

In order to further understand the origin of the superior HER activity of the TiO_2_@Ni_3_S_2_ core/branch nanoflake arrays, the effective electrochemical active surface area (ECSA) of the three samples was calculated by determining the double-layer capacitance (*C*_dl_) based on the CV results at different scan rates (Fig. S6b–d). The obtained current density is plotted as a function of scan rates in Fig. 4c. The ECSA value is considered to be linearly proportional to the *C*_dl_ value, equaling half the slope value. Notably, the TiO_2_@Ni_3_S_2_ electrode possesses a high capacitance, up to 42 mF cm^−2^, which is much higher than those of Ni_2_(OH)_2_CO_3_ (24 mF cm^−2^) and Ni_3_S_2_ (29 mF cm^−2^) electrodes. The above results indicate that the TiO_2_@Ni_3_S_2_ electrode has more exposed active sites. EIS tests were performed to further probe the electrochemical behavior during the HER process. Figure 4d exhibits the Nyquist plots of all electrodes. The semicircle represents the charge transfer resistance (*R*_ct_) of the hydrogen evolution reaction. Remarkably, the TiO_2_@Ni_3_S_2_ electrode shows the smallest *R*_ct_ value among the three electrodes, which suggests that it facilitates the fastest dynamics during HER. Moreover, the solution resistance (*R*_s_) values of Ni_2_(OH)_2_CO_3_, Ni_3_S_2_, and TiO_2_@Ni_3_S_2_ electrodes are 1.49, 1.46, and 1.45 Ω, respectively. These findings further verify that TiO_2_@Ni_3_S_2_ still possesses high electronic conductivity and charge transfer ability during the entire hydrogen evolution reaction. In addition, the TiO_2_@Ni_3_S_2_ electrode also shows long-term durability with no decay after 10 h at a large current density of 100 mA cm^−2^ (Fig. 4e).

**Fig. 5:**
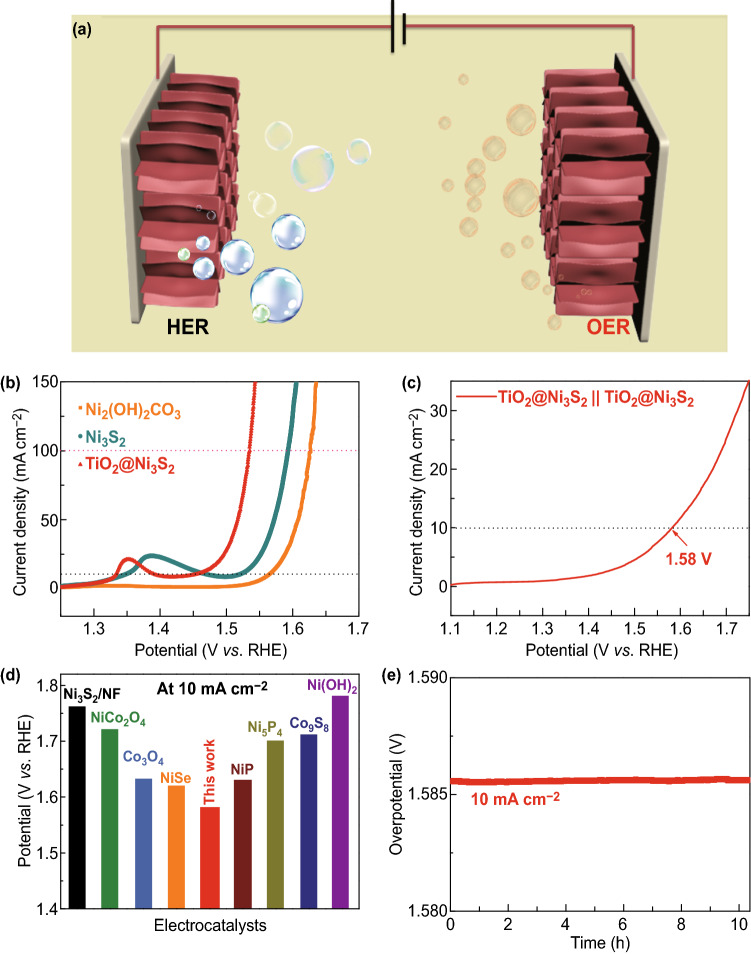
**a** Schematic illustration of the overall water-splitting process using the bifunctional electrocatalyst. **b** LSV curves at 5 mV s^−1^ for the OER performances using the Ni_2_(OH)_2_CO_3_, Ni_3_S_2_, and TiO_2_@Ni_3_S_2_ electrodes. **c** LSV curves of the overall water-splitting performance of the TiO_2_@Ni_3_S_2_||TiO_2_@Ni_3_S_2_ electrolyzer. **d** Comparison of the overall water-splitting performance of our TiO_2_@Ni_3_S_2_||TiO_2_@Ni_3_S_2_ electrolyzer with those of other electrocatalysts in the literature, and **e** electrochemical stability at 10 mA cm^−2^

As shown in Fig. 5a, the electrolysis cell of the two-electrode system consists of the TiO_2_@Ni_3_S_2_ electrocatalyst as both anode and cathode in 1 M KOH solution (denoted as TiO_2_@Ni_3_S_2_ || TiO_2_@Ni_3_S_2_). Apart from the outstanding HER activity, the as-prepared TiO_2_@Ni_3_S_2_ electrode also delivers excellent OER catalytic performance in the alkaline solution.

As shown in Fig. 5b, the TiO_2_@Ni_3_S_2_ electrode exhibits a remarkably low OER overpotential of 220 mV at 10 mA cm^−2^ and 300 mV at 100 mA cm^−2^, superior to those of the Ni_2_(OH)_2_CO_3_ (330 mV, 390 mV) and Ni_3_S_2_ (280 mV, 360 mV) electrodes. Owing to its excellent catalytic activities in both OER and HER, the TiO_2_@Ni_3_S_2_ electrode could be utilized as an attractive bifunctional electrocatalyst for water splitting in an alkaline medium. Impressively, a noticeably low cell voltage of 1.58 V is gained at the current density of 10 mA cm^−2^ (Fig. 5c), better than those of the other reported bifunctional electrocatalysts (Fig. 5d) [1, 9, 26, 43–47]. In order to investigate the change in the chemical composition of TiO_2_@Ni_3_S_2_, high-resolution Ni 2*p* spectra were acquired after HER and OER tests (Fig. S7). After HER tests, the Ni 2*p* spectrum remained almost the same as before with a slight redshift owing to the cathodic H_2_ reduction. However, after the OER test, the peak at 853.08 eV disappeared and the intensity of the satellite peak (2p_3/2_) decreased because of the formation of hydrated nickel oxide. Furthermore, the TiO_2_@Ni_3_S_2_ || TiO_2_@Ni_3_S_2_ catalyzer cell shows long-term durability with no decay after 10 h (Fig. 5c, e). All the above results demonstrate that the TiO_2_@Ni_3_S_2_ core/branch arrays possess superior electrochemical activity in both HER and OER, suggesting that the designed TiO_2_@Ni_3_S_2_ core/branch arrays would be promising electrocatalysts for practical application in alkaline water splitting.

## Conclusion

We developed a facile and high-efficiency low-temperature sulfurization method for the large-scale synthesis of novel binder-free TiO_2_@Ni_3_S_2_ core/branch arrays. Impressively, the as-obtained Ni_3_S_2_ nanoflake branches exposed the highly active {$\bar{2}10$} high-index facet. Strong support and induced directional growth of Ni_3_S_2_ nanoflakes are realized with the aid of the ALD-TiO_2_ skeleton. Owing to large surface area of the core/branch arrays, large porosity, and binder-free adhesion as well as richer active sites of the exposed {$\bar{2}10$} high-indexed facets of Ni_3_S_2_ nanoflakes, the designed TiO_2_@Ni_3_S_2_ core/branch arrays deliver low overpotentials and Tafel slopes for both OER and HER as well as cycling stability in an alkaline medium superior to those of the other Ni_3_S_2_ counterparts. Our work offers a facile low-temperature strategy to construct advanced metal sulfide catalysts for electrochemical energy conversion and storage.

## Electronic supplementary material

Below is the link to the electronic supplementary material.

Supplementary material 1 (PDF 969 kb)
