# Correction to: High-Index-Faceted Ni_3_S_2_ Branch Arrays as Bifunctional Electrocatalysts for Efficient Water Splitting

**DOI:** 10.1007/s40820-020-00530-1

**Published:** 2020-10-31

**Authors:** Shengjue Deng, Kaili Zhang, Dong Xie, Yan Zhang, Yongqi Zhang, Yadong Wang, Jianbo Wu, Xiuli Wang, Hong Jin Fan, Xinhui Xia, Jiangping Tu

**Affiliations:** 1 State Key Laboratory of Silicon Materials, Key Laboratory of Advanced Materials and Applications for Batteries of Zhejiang Province, and Department of Materials Science and Engineering, Zhejiang University, Hangzhou, 310027 People’s Republic of China; 2 Guangdong Engineering and Technology Research Center for Advanced Nanomaterials, School of Environment and Civil Engineering, Dongguan University of Technology, Dongguan, 523808 People’s Republic of China; 3 School of Physical and Mathematical Sciences, Nanyang Technological University, Singapore, 637371 Singapore; 4 School of Engineering, Nanyang Polytechnic, Singapore, 569830 Singapore; 5 Zhejiang Provincial Key Laboratory for Cutting Tools, Taizhou University, Taizhou, 318000 People’s Republic of China

## Correction to: Nano-Micro Lett. (2019) 11:12 10.1007/s40820-019-0242-8

In the original publication, Figure S4 is an ancillary image to compare the specific surface areas of TiO_2_/Ni_3_S_2_ and Ni_3_S_2_ samples and it was incorrectly published. To better serve our readers, the correct figure is provided in this correction. The BET values are correct and unaffected. The corresponding figure caption, data analysis and conclusions are not affected and thus not to be changed. The authors would like to apologize for any inconvenience caused.
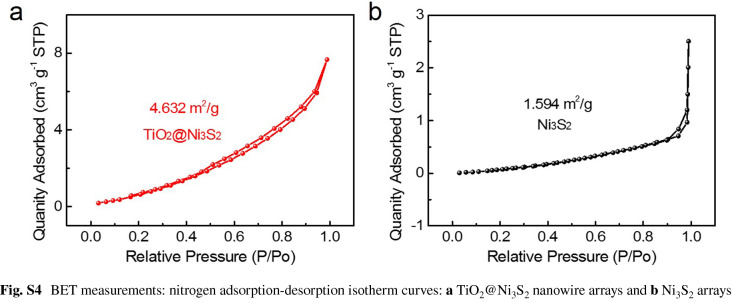


## Electronic supplementary material

Below is the link to the electronic supplementary material.

Supplementary material 1 (JPG 95 kb)

