# Supplementary material for: High-Index-Faceted Ni3S2 Branch Arrays as Bifunctional Electrocatalysts for Efficient Water Splitting
Source: Nanomicro Lett. 2019 Feb 5;11:12. doi: 10.1007/s40820-019-0242-8 (PMC7770978; doi:10.1007/s40820-019-0242-8)
Supplement: Supplementary file 1 — Supplementary material 1 (PDF 969 kb) [file 40820_2019_242_MOESM1_ESM.pdf]

Supporting Information for

High-Index-Faceted Ni_3S_2 Branch Arrays as Bifunctional Electrocatalysts for Efficient Water Splitting

Shengjue Deng[1, ‡], Kaili Zhang[1, ‡], Dong Xie[2], Yan Zhang[1], Yongqi Zhang[3], Yadong Wang[4], Jianbo Wu[5], Xiuli Wang[1], Hong Jin Fan[3], Xinhui Xia[1, *], Jiangping Tu[1, *]

[1]State Key Laboratory of Silicon Materials, Key Laboratory of Advanced Materials and Applications for Batteries of Zhejiang Province, and Department of Materials Science and Engineering, Zhejiang University, Hangzhou 310027, People's Republic of China

[2]Guangdong Engineering and Technology Research Center for Advanced Nanomaterials, School of Environment and Civil Engineering, Dongguan University of Technology, Dongguan 523808, People's Republic of China

[3]School of Physical and Mathematical Sciences, Nanyang Technological University, 637371, Singapore

[4]School of Engineering, Nanyang Polytechnic, 569830, Singapore

[5]Zhejiang Provincial Key Laboratory for Cutting Tools, Taizhou University, Taizhou 318000, People's Republic of China

‡Shengjue Deng and Kaili Zhang contributed equally to this work

*Corresponding authors. E-mail: helloxxh@zju.edu.cn (Xinhui Xia); tujp@zju.edu.cn (Jiangping Tu)

Supplementary Figures

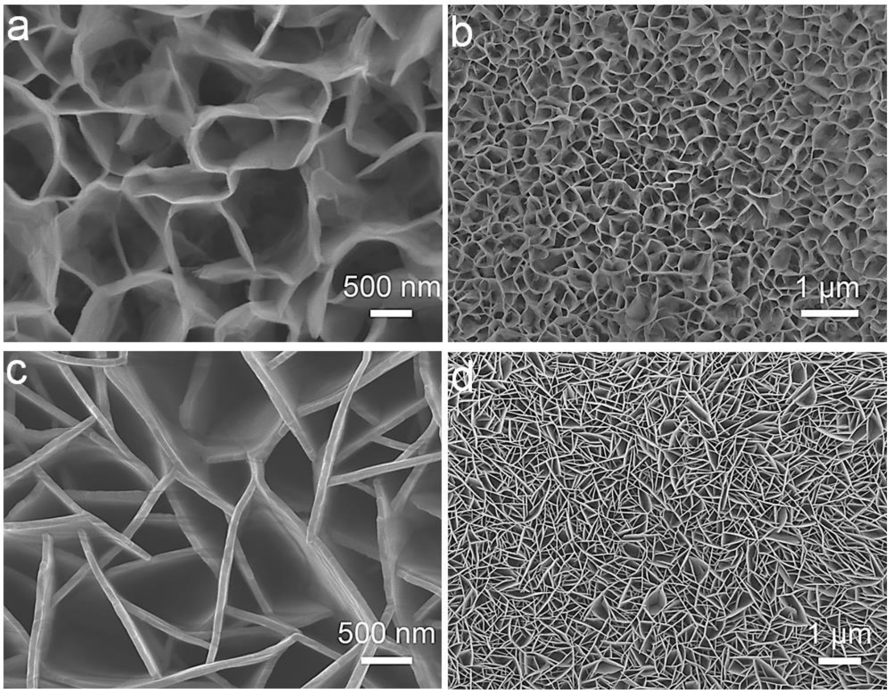

Fig. S1 SEM images of $Ni_2(OH)_2CO_3$ and $TiO_2@Ni_2(OH)_2CO_3$ nanoflake arrays

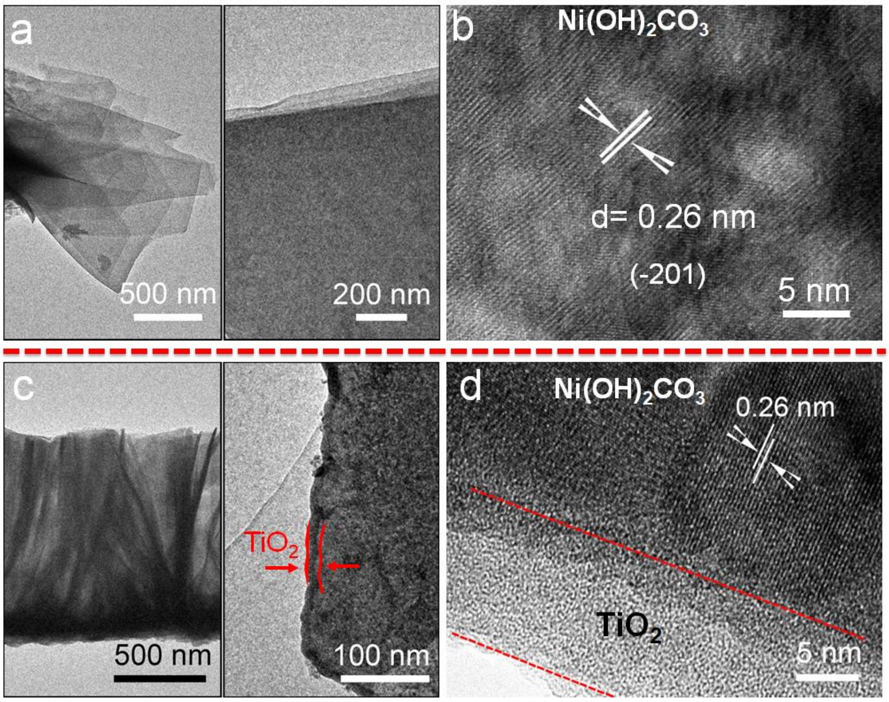

Fig. S2 TEM images of $Ni_2(OH)_2CO_3$ and $TiO_2@Ni_2(OH)_2CO_3$ nanoflakes

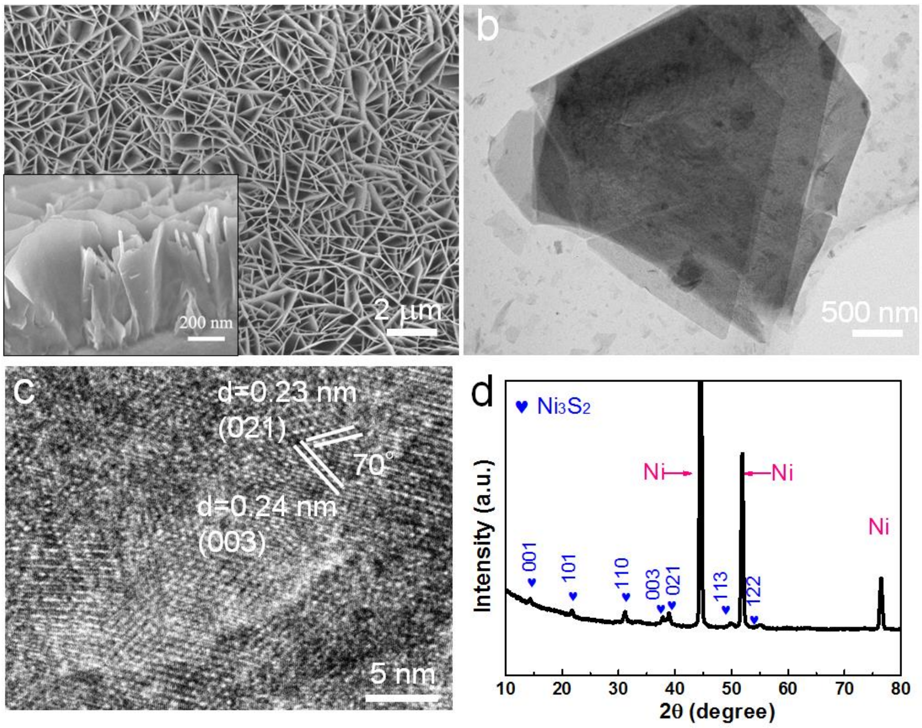

Fig. S3 Morphology and structure of control sample of pure Ni_3S_2 nanoflake arrays prepared without the TiO_2 support: **a** SEM image (fine structure in inset); **b, c** TEM-HRTEM images; **d** XRD pattern

SEM image (**Fig. S3**a) presents the uniform distribution of Ni_3S_2 nanoflakes on the nickel foam. TEM-HRTEM images (**Fig. S3**b, c) show the formation of regular Ni_3S_2 nanoflakes. When the HRTEM image is performed along the [100] crystallographic direction of Ni_3S_2, the (003) and (021) plane as well as the interior angle between the (003) and (021) facets of ~70° can be still detected, which is also corresponding with the exposed $\{\bar{2}10\}$ facet. XRD pattern further suggests the successful preparation of Ni_3S_2 (JCPDS 44-1418) **(Fig. S3**d).

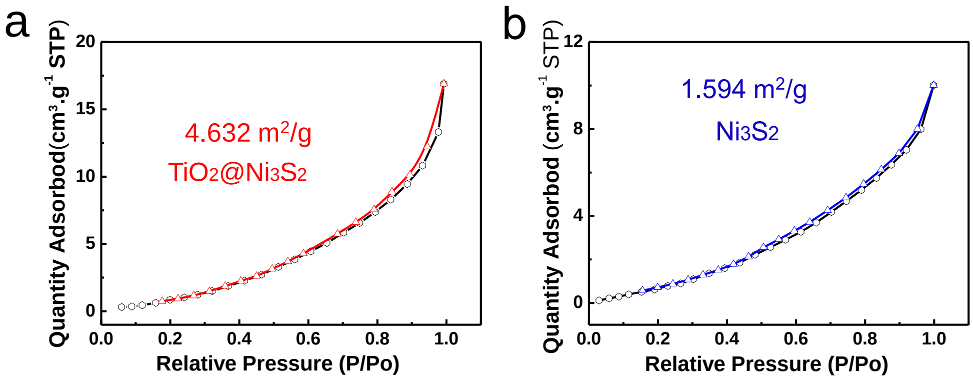

Fig. S4 BET measurements: nitrogen adsorption-desorption isotherm curves: **a** TiO_2 @Ni_3S_2 nanowire arrays and **b** Ni_3S_2 arrays

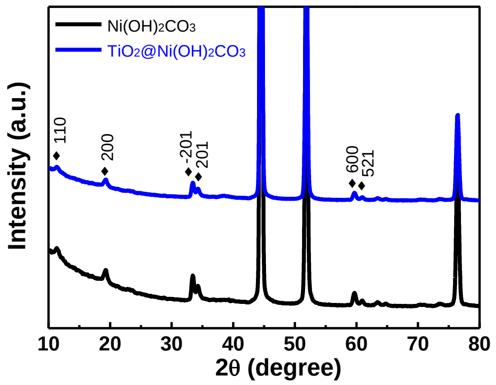

Fig. S5 XRD pattern of $Ni_2(OH)_2CO_3$ and $TiO_2@Ni_2(OH)_2CO_3$ arrays

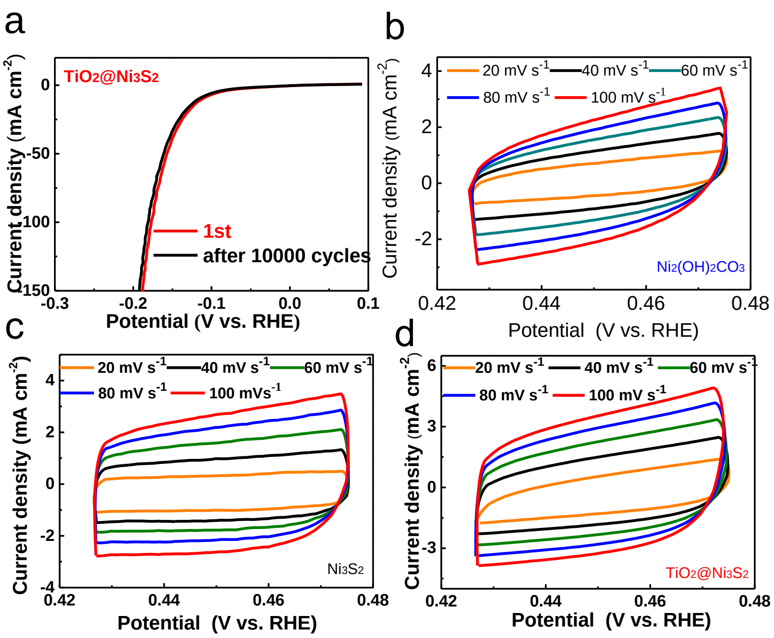

Fig. S6 a LSV plots for HER of the $TiO_2@Ni_3S_2$ electrode in the first and after 10,000 cycles. CV curves of different electrodes in double layer region at various scan rates: **b** $Ni_2(OH)_2CO_3$, **c** Ni_3S_2, and **d** $TiO_2@Ni_3S_2$ electrodes

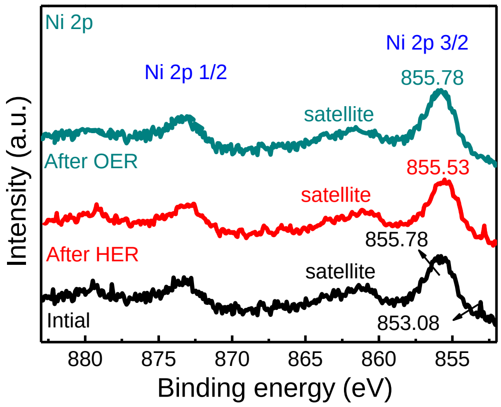

Fig. S7 XPS spectra of Ni 2p: **a** Initial state, **b** After HER and **c** After OER tests
