# Supplementary figures and images for: Correction to: High-Index-Faceted Ni3S2 Branch Arrays as Bifunctional Electrocatalysts for Efficient Water Splitting
Source: Nanomicro Lett. 2020 Oct 31;13:16. doi: 10.1007/s40820-020-00530-1 (PMC8187692; doi:10.1007/s40820-020-00530-1)

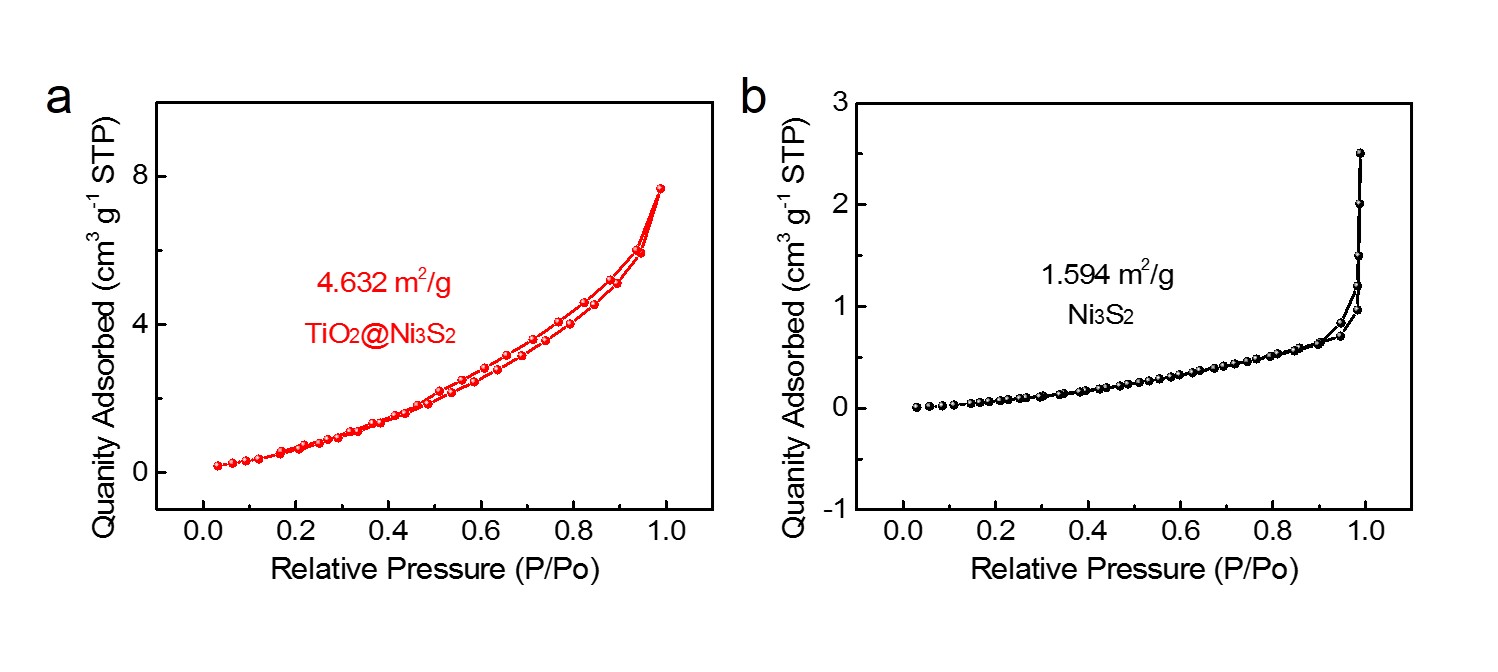

Supplement: Supplementary file 1 — Supplementary material 1 (JPG 95 kb) [file 40820_2020_530_MOESM1_ESM.jpg]
